# Immunofibrotic drivers of impaired lung function in postacute sequelae of SARS-CoV-2 infection

**DOI:** 10.1172/jci.insight.148476

**Published:** 2021-07-22

**Authors:** Hyung J. Chun, Elias Coutavas, Alexander B. Pine, Alfred I. Lee, Vanessa L. Yu, Marcus K. Shallow, Coral X. Giovacchini, Anne M. Mathews, Brian Stephenson, Loretta G. Que, Patty J. Lee, Bryan D. Kraft

**Affiliations:** 1 Yale Cardiovascular Research Center, Section of Cardiovascular Medicine, Department of Internal Medicine, Yale School of Medicine, New Haven, Connecticut, USA.; 2 Division of Pulmonary, Allergy, and Critical Care Medicine, Department of Medicine, Duke University School of Medicine, Durham, North Carolina, USA.; 3 Section of Hematology, Department of Internal Medicine, Yale School of Medicine, New Haven, Connecticut, USA.

**Keywords:** COVID-19, Pulmonology, Fibrosis, Growth factors, Neutrophils

## Abstract

**BACKGROUND:**

Individuals recovering from COVID-19 frequently experience persistent respiratory ailments, which are key elements of postacute sequelae of SARS-CoV-2 infection (PASC); however, little is known about the underlying biological factors that may direct lung recovery and the extent to which these are affected by COVID-19 severity.

**METHODS:**

We performed a prospective cohort study of individuals with persistent symptoms after acute COVID-19, collecting clinical data, pulmonary function tests, and plasma samples used for multiplex profiling of inflammatory, metabolic, angiogenic, and fibrotic factors.

**RESULTS:**

Sixty-one participants were enrolled across 2 academic medical centers at a median of 9 weeks (interquartile range, 6–10 weeks) after COVID-19 illness: n = 13 participants (21%) had mild COVID-19 and were not hospitalized, n = 30 participants (49%) were hospitalized but were considered noncritical, and n = 18 participants (30%) were hospitalized and in the intensive care unit (ICU). Fifty-three participants (85%) had lingering symptoms, most commonly dyspnea (69%) and cough (58%). Forced vital capacity (FVC), forced expiratory volume in 1 second (FEV1), and diffusing capacity for carbon monoxide (DLCO) declined as COVID-19 severity increased (*P <* 0.05) but these values did not correlate with respiratory symptoms. Partial least-squares discriminant analysis of plasma biomarker profiles clustered participants by past COVID-19 severity. Lipocalin-2 (LCN2), MMP-7, and HGF identified by our analysis were significantly higher in the ICU group (*P <* 0.05), inversely correlated with FVC and DLCO (*P <* 0.05), and were confirmed in a separate validation cohort (*n =* 53).

**CONCLUSION:**

Subjective respiratory symptoms are common after acute COVID-19 illness but do not correlate with COVID-19 severity or pulmonary function. Host response profiles reflecting neutrophil activation (LCN2), fibrosis signaling (MMP-7), and alveolar repair (HGF) track with lung impairment and may be novel therapeutic or prognostic targets.

**Funding:**

National Heart, Lung, and Blood Institute (K08HL130557 and R01HL142818), American Heart Association (Transformational Project Award), the DeLuca Foundation Award, a donation from Jack Levin to the Benign Hematology Program at Yale University, and Duke University.

## Graphical abstract

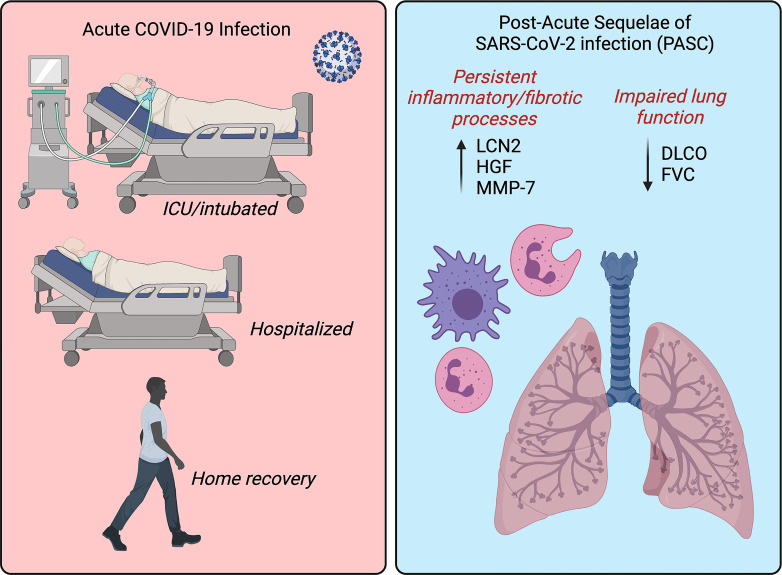

## Introduction

As the number of patients who recover from acute coronavirus disease 2019 (COVID-19) rises, it is increasingly apparent that a substantial subset displays persistent subjective and objective respiratory ailments. Patients report a high prevalence of lingering respiratory symptoms, such as fatigue and dyspnea (1–4), and spirometry and diffusion capacity testing show impaired pulmonary function (3). However, little is known about the biological drivers of long-term respiratory disease after COVID-19 and how acute COVID illness severity affects the emerging COVID-19 long-hauler syndrome or postacute sequelae of SARS-CoV-2 infection (PASC). To better understand the relationship between subjective and objective respiratory abnormalities and underlying biological drivers, we measured symptom burden, pulmonary function tests, and plasma biomarkers in individuals that have recovered from COVID-19 infection.

## Results

**Figure 1:**
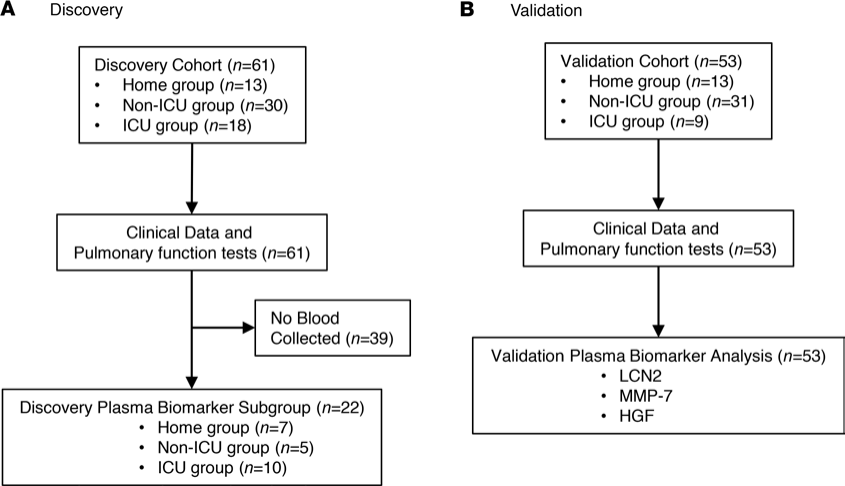
CONSORT diagrams of the study populations. CONSORT diagram of subjects enrolled in the (**A**) discovery cohort and (**B**) validation cohort.

We enrolled 61 individuals into the post–COVID-19 clinics (Figure 1). Subject demographics for each group are shown in Table 1. Thirteen individuals (21%) recovered at home (referred to as the home group), thirty individuals (48%) were hospitalized but did not require the intensive care unit (ICU) (referred to as the non-ICU group), and nineteen individuals (31%) were hospitalized and required ICU-level care (referred to as the ICU group). Median (IQR) age was 53 years (43–62 years) and sex distribution was equitable: 56% male and 44% female participants. Median (IQR) time from onset of COVID-19 infection to post-COVID follow-up was 9 weeks (6–11 weeks) but was not significantly different among the 3 COVID-19 groups. Eight of the subjects admitted to the ICU required intubation and mechanical ventilation, but none required tracheostomy. The demographics and characteristics of the subgroup of individuals that underwent plasma biomarker testing are also shown (Table 1).

**Figure 2:**
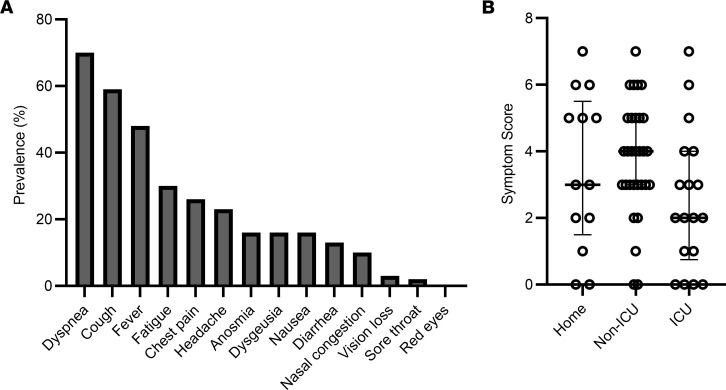
Symptom burden after COVID-19. (**A**) Prevalence of symptoms reported by participants (*n =* 61) at the time of initial follow-up visit. (**B**) Symptom scores (total number of symptoms reported), with median bars and interquartile range error bars for the home (*n =* 13), non-ICU (*n =* 30), and ICU (*n =* 18) groups. There were no significant differences between groups using the Kruskal-Wallis test with Benjamini-Hochberg post hoc test.

**Figure 3:**
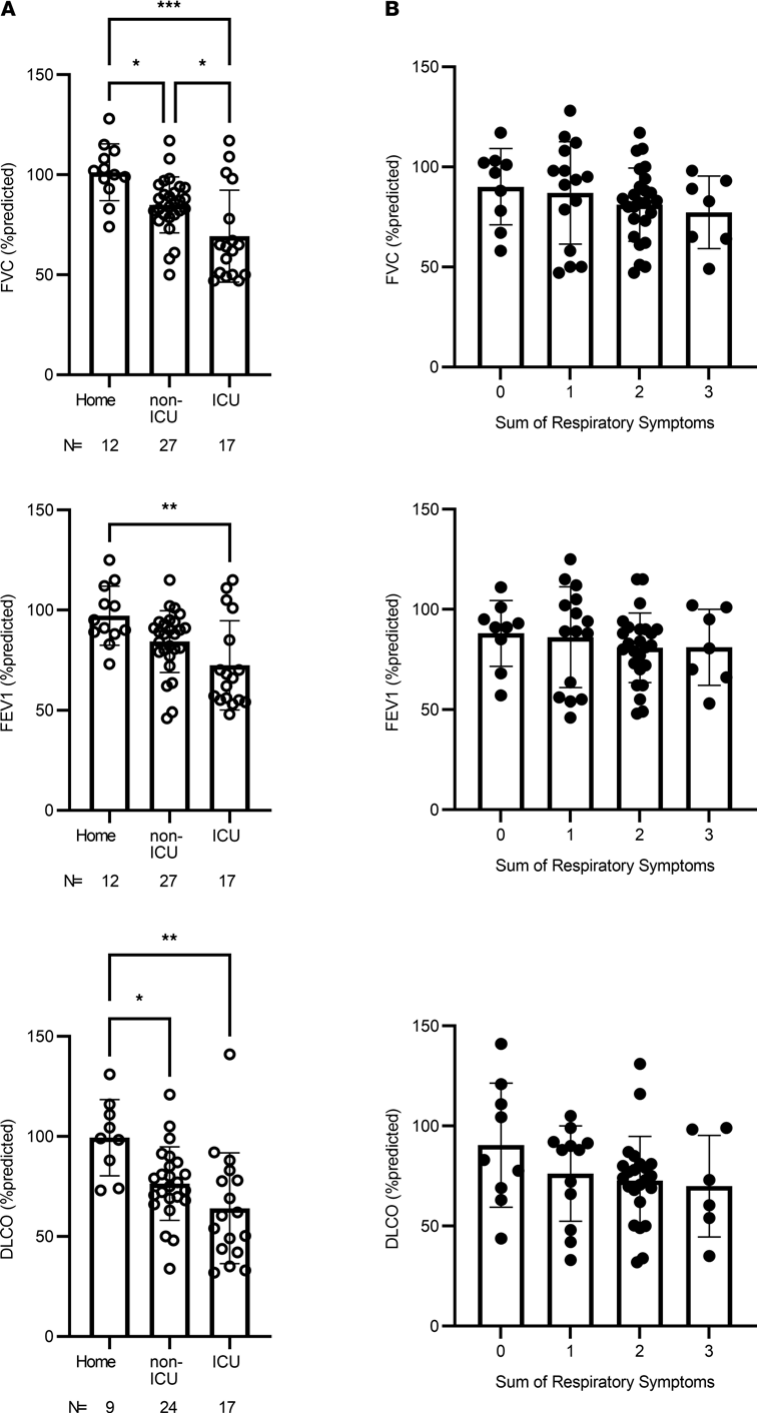
Pulmonary function tests and respiratory symptom score. (**A**) Scatter plots show forced vital capacity (FVC), forced expiratory volume in 1 second (FEV1), and diffusing capacity for carbon monoxide (DLCO) as percentages of predicted values for each participant in the home, non-ICU, and ICU groups for the number (*n*) shown. (**B**) Scatter plots show the sum of respiratory symptoms (1 point each for dyspnea, cough, and fatigue) vs. FVC, FEV1, and DLCO. Bars represent medians, and error bars represent the interquartile range. Statistical analysis by Kruskal-Wallis test with Benjamini-Hochberg post hoc test. *P < 0.05, **P < 0.01, ***P < 0.001.

Fifty-three individuals (85%) reported lingering symptoms of COVID-19, the most common being dyspnea (69%), cough (58%), fever (47%), fatigue (29%), chest pain (26%), and headache (23%; Figure 2A). There were no significant differences in symptom burden among the home, non-ICU, and ICU groups (*P* = NS; Figure 2B). Pulmonary function testing was performed on 59 participants. Forced vital capacity (FVC), forced expiratory volume in 1 second (FEV1), and diffusing capacity for carbon monoxide (DLCO) decreased as severity of COVID-19 increased (Figure 3A). There was no relationship between pulmonary function and presence of persistent respiratory symptoms, measured by the sum of the scores for fatigue, dyspnea, and cough (Figure 3B).

**Figure 4:**
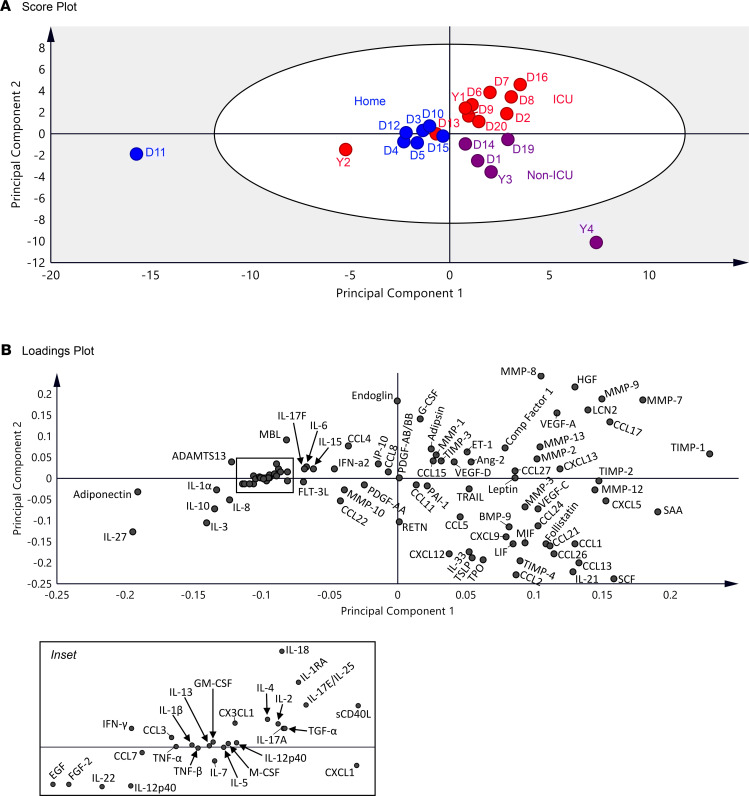
Partial least-squares discriminant analysis. (**A**) The score plot shows each individual in the home (blue circles, *n =* 7), non-ICU (purple circles, *n =* 5), and ICU (red circles, *n =* 10) groups. The gray zone in the score plot depicts outliers with 95% confidence. The Q2 value for 2 components is 0.22788. (**B**) The loadings plot depicts associations between clusters and the measured biomarkers.

To identify potential biological factors that associate with impaired pulmonary function after COVID-19 infection, we measured levels of circulating inflammatory, metabolic, angiogenic, and fibrotic markers in plasma from a subset of 22 participants (home group, *n =* 7; non-ICU group, *n =* 5; ICU group, *n =* 10) and performed partial least-squares discriminant analysis (PLS-DA; Figure 4). The score plot (Figure 4A) shows that participants largely cluster in distinct quadrants based on how severe their COVID-19 illness was, even approximately 9 weeks after infection. In fact, the clustering shows a counterclockwise rotation around the origin as severity of viral infection increases from home to non-ICU to ICU-level of care. The Q2 value for 2 components is 0.22788. The loadings plot (Figure 4B) shows the associations between the clusters of participants and the measured plasma biomarkers (Supplemental Table 1; supplemental material available online with this article; https://doi.org/10.1172/jci.insight.148476DS1). The top right quadrant shows the biomarkers that directly correlate with critical COVID-19 illness requiring ICU admission. Several of the most impactful biomarkers (i.e., furthest from the point of origin) that associate with ICU level of care included MMPs (MMP-7, MMP-8, and MMP-9), markers of immune activation (lipocalin-2 [LCN2], HGF, and CCL17; Figure 4B). Plasma levels of all 3 factors were similar between the control and home groups but increased with COVID-19 severity and were significantly higher in the ICU group.

**Figure 5:**
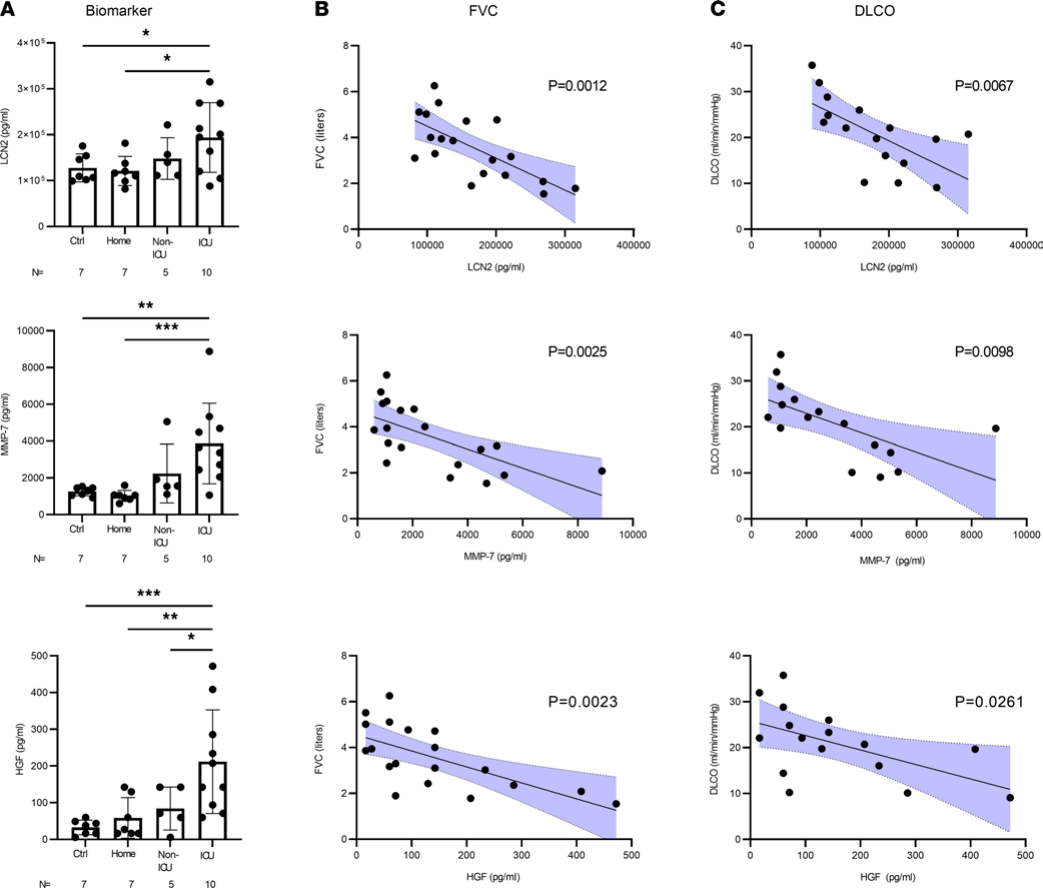
Biomarkers associated with impaired pulmonary function after COVID-19 (A) Plasma levels of biomarkers lipocalin-2 (LCN2), MMP-7, and HGF in individuals (black circles) in the control (Ctrl), home, non-ICU, and ICU groups. The top of each graph column represents the mean, and error bars represent standard deviation. Statistical analysis by 1-way ANOVA with Fisher’s LSD post hoc test. *P < 0.05, **P < 0.01, ***P < 0.001. (**B** and **C**) Scatter plots for individuals (black circles) of LCN2, MMP-7, and HGF on abscissa versus forced vital capacity (FVC) or diffusing capacity (DLCO) on ordinate. Statistical analysis is simple linear regression. Blue shaded area shows 95% confidence intervals.

To further determine if specific circulating factors are associated with persistently impaired pulmonary function, we used random-forest feature selection (5) to identify which factors best explain the variability seen in FVC and DLCO (Supplemental Figures 1 and 2). This analysis identified MMP-7, LCN2, HGF, and leptin as the markers that were most significantly associated with FVC and DLCO. In our patient cohort, leptin did not differ by acute COVID-19 severity but was strongly inversely correlated with post–COVID-19 FVC and also directly correlated with body mass index (Supplemental Figure 3). However, after adjusting for body mass index, the relationship between leptin and FVC was no longer significant. We further evaluated LCN2, MMP-7, and HGF, as these were the most significantly associated with FVC and DLCO. Interestingly, we found that the plasma levels of LCN2, MMP-7, and HGF were higher at follow-up in patients with more severe acute COVID-19 illness (Figure 5A). Confirmation of the relationship between LCN2, MMP-7, and HGF and pulmonary function using simple linear regression identified a highly significant, inverse correlation with FVC and DLCO (Figure 5, B and C). There was no relationship between these 3 biomarkers and burden of subjective symptoms.

To validate these 3 biomarkers, we measured them in a separate group of 53 participants (referred to as the validation cohort; Figure 1, Table 1, and Supplemental Figure 4A). The validation cohort consisted of *n =* 13 participants in the home group, *n =* 31 participants in the hospitalized, non-ICU group, and *n =* 9 participants in the ICU group. Demographics of the discovery and validation cohorts were comparable (Table 1). Plasma levels of LCN2 and MMP-7 were significantly higher in the validation ICU group compared with the validation home group, whereas a statistical trend was observed for HGF (Supplemental Figure 4A). However, like the discovery cohort, all 3 biomarkers were highly linearly correlated with decline in FVC and DLCO (Supplemental Figure 4, B and C).

## Discussion

Herein, we report that among patients returning for follow-up visits for persistent symptoms after their acute COVID-19 illness, respiratory symptoms and impaired lung function are common. Pulmonary function in particular worsens as COVID-19 illness severity increases. We further show that plasma biomarker profiles measured at 9 weeks after COVID-19 infection reveal prior COVID-19 severity and cluster patients accurately. Plasma LCN-2, MMP-7, and HGF levels are highest in survivors of severe COVID-19 infection and correlate strongly with pulmonary function impairment.

Patients recovering from acute COVID-19 infection commonly display persistent symptoms such as fatigue and dyspnea (1–4). Our study found a high degree of symptom burden across subgroups (home, non-ICU, and ICU) that did not associate by COVID-19 severity (3). Impaired pulmonary function was reported by Huang et al. (1) and, similar to our findings, correlated with prior COVID-19 severity. Together, these data highlight the discordance of subjective and objective respiratory abnormalities after COVID-19 infection (3), a well-known phenomenon in cardiopulmonary diseases in general (6), and indicate that subjective symptoms alone cannot discern degree of post–COVID-19 lung function impairment.

Partial least-squares discriminant analysis could identify and separate subjects by COVID-19 illness severity based on their plasma biomarker profiles, even 9 weeks after recovery. Our analysis highlighted a number of important biomarkers that strongly associated with severe/ICU COVID-19 status. We chose to further analyze 3 of these markers, LCN2, MMP-7, and HGF, based on their differing roles in host response. While these markers were similar between the healthy controls and the home group, there was a stepwise increase seen as COVID-19 severity increased. Furthermore, LCN2, MMP-7, and HGF were not associated with subjective symptoms in this study, and have not been previously reported to be associated with subjective symptoms, but were strongly associated with objective respiratory function. These findings are confirmed by the validation cohort, which also found strong linear relationships between plasma biomarker levels and decline in respiratory function. LCN2 is a component of neutrophil secondary granules that was found to strongly associate with acute COVID-19 critical illness and mortality (7–10); however, the role in post–COVID-19 recovery has not been previously described. That higher levels of LCN-2 are found at 9 weeks after severe COVID-19 illness compared with that in nonsevere cases suggests these patients may have ongoing neutrophil activation that could be amenable to targeted therapy.

MMP-7 is a protease that breaks down extracellular matrix deposited in the lung after injury. The role of MMP-7 in COVID-19 has not been reported; however, previous studies of non–COVID-19 acute lung injury have shown higher circulating levels of MMPs (11) that associate with worse disease severity and clinical outcomes (12). MMP-7 is also a validated biomarker for idiopathic pulmonary fibrosis severity that correlates with pulmonary function (13), and our findings suggest that MMP-7 may be a novel biomarker of COVID-19 ARDS recovery.

HGF is a growth factor secreted by alveolar fibroblasts shown to be elevated in bronchoalveolar lavage fluid of patients with ARDS (14) and in plasma of patients with acute COVID-19 illness (15); however, the plasma levels after COVID-19 recovery have not been previously reported. In vivo and in vitro studies indicate that HGF promotes alveolar epithelial and endothelial repair after acute lung injury (16, 17) via induction of counterinflammatory and antioxidant gene expression (18–20). Our findings suggest that high HGF levels at 9 weeks post–COVID-19 could indicate ongoing alveolar repair proportionate to the degree of acute lung injury; however, further studies are needed to determine the effect of HGF on COVID-19 recovery.

Leptin is a hormone secreted by adipocytes in proportion to obesity, a risk factor for severe acute COVID-19 (21), and may contribute to immune dysregulation during acute COVID-19 (22, 23). In our patient population with post–COVID-19 syndrome, we found that leptin was inversely related to FVC; however, after adjusting for body mass index, the relationship was no longer significant, indicating body mass index was a confounder. Future studies should take body mass index into account when reporting leptin results.

Multiple studies have implicated hypercoagulable state, driven by factors such as endotheliopathy, complement pathway activation, and platelet activation, as having an important role in severity of acute illness in COVID-19 (24–26). In our study, we did not identify a meaningful association among levels of biomarkers that are associated with these pathologic processes and persistence of respiratory symptoms. An assessment of a possible role of these processes and their contribution to other nonrespiratory symptoms and clinical findings of PASC will be needed.

Our study has several limitations. First, we only recruited individuals that had ongoing symptoms after COVID-19 infection; therefore, we cannot report the true prevalence of symptoms or pulmonary function derangements in individuals with asymptomatic infection or in individuals with symptom resolution after COVID-19 infection. Second, our plasma biomarker profiles represent a single time point for each individuals, and therefore, we cannot draw conclusions about trajectory or changes over time. Third, we cannot establish a mechanism in this human cohort study; however, our findings are highly associational and lay the foundations to drive further work in this area. Fourth, the PLS-DA model was at risk for being overfit, consistent with a modest Q2 value of only approximately 0.2. However this was mitigated with similar findings of the Boruta algorithm and addressed with the addition of the validation cohort, which confirmed the findings of the discovery cohort. Finally, the majority of the patients were assessed at 9 weeks after their acute COVID-19 infection, which may not provide sufficient insights into the long-term lung repair that is likely to occur beyond this time frame. Future studies evaluating biomarker profiles and pulmonary function at later time points will provide greater insights into persistent symptoms associated with COVID-19 illness.

In conclusion, persistent respiratory symptoms and impaired lung function are common after acute COVID-19 illness, but subjective symptoms alone do not predict lung injury. Partial least-squares discriminant analysis identifies and separates individuals by COVID-19 illness severity based on their plasma biomarker profiles, even 9 weeks after recovery. Circulating factors associated with acute neutrophil activation, fibrosis signaling, and alveolar epithelial repair remain elevated in survivors of acute COVID-19 infection and strongly associate with impaired pulmonary function. Our study provides potentially novel insights into divergent host responses of lung repair after COVID-19 pneumonia and suggest that immunologic and fibrotic drivers of disease severity in the acute COVID-19 illness may regulate the trajectory of recovery in the post–COVID-19 phase. These markers may represent key prognostic tools that can be followed during and after COVID-19 illness and may identify COVID-19 survivors at increased risk for chronic parenchymal lung disease. These findings will require further validation in larger studies.

## Methods

### Data collection.

All participants completed symptom questionnaires (presence or absence of the following: fatigue, fever, headache, dyspnea, cough, chest pain, nausea, diarrhea, anosmia, dysgeusia, sore throat, nasal congestion, red eyes, and vision loss). All participants underwent physical examinations and pulmonary function testing (spirometry and diffusion capacity) at the time of their follow-up visits. Symptom scores were calculated by the sum of the number of symptoms reported. Respiratory symptom scores were calculated by the sum of the following symptoms: cough, shortness of breath, and fatigue. Severity of COVID-19 infection was defined by need for ICU hospital care, non-ICU hospital care (non-ICU), or nonhospitalized home recuperation (home). Whole blood was collected by venipuncture, centrifuged, separated into plasma, and stored at –80°C until use.

### Plasma biomarker profiling.

Multiplex profiling of plasma was performed on a subset of discovery subjects (*n =* 22) and a validation cohort (*n =* 53) by Eve Technologies using the following assays: Human Cytokine 71-Plex, Human Complement Panel 1, Human Angiogenesis 17-Plex, Human MMP 9-Plex and TIMP 4-Plex, and Human Adipokine 5-Plex. A complete list of biomarkers profiled is provided in Supplemental Table 1.

### Statistics.

Grouped data are expressed as median (interquartile range) unless specified otherwise. Differences between groups were analyzed by Kruskal-Wallis test with Benjamini-Hochberg post hoc test to control for false discovery rate or 1-way ANOVA with Fisher’s LSD post hoc test (Graphpad Prism). Simple linear regression and multivariate linear regression were performed by Prism. Skewed data sets were log transformed for normality. PLS-DA was performed using SIMCA v15 (Umetrics), and the Q2 value was reported using MetaboAnalyst 5.0 (https://www.metaboanalyst.ca) (27). The random-forest feature selection (Boruta algorithm) was performed using the Boruta package in R software. All *P* values are 2 tailed. *P <* 0.05 was accepted as statistically significant. Statistical trends were noted for *P <* 0.1.

### Study approval.

The studies were approved by the institutional review boards of Yale University (no. 2000027792) and Duke University (Pro00105518 and Pro00106151). Participants with persistent symptoms 30 days after the diagnosis of acute COVID-19 infection were considered eligible and recruited to a post–COVID-19 clinic for further evaluation. Seven asymptomatic, nonhospitalized healthy control subjects were enrolled at Yale under a separate approved protocol (no. 1401013259) (median age 45 years [interquartile range 44–53 years]; 2 male and 5 female participants). The discovery group participants were enrolled between June and August 2020, and validation group participants were enrolled from September 2020 to March 2021. Participants were selected for validation group that had both pulmonary function tests and blood samples collected at the initial study visit at 6–9 weeks after COVID infection. Written informed consent was provided by all individuals prior to study activities.

## Author contributions

HJC and BDK wrote the manuscript, which was approved by all authors. EC, ABP, VLY, MKS, CXG, AMM, and BS participated in data collection, analysis, and/or interpretation. The overall study design was conceived by HJC, BDK, LGQ, AIL, and PJL.

## Supplementary Material

Supplemental data

Supplemental Data Set 1

## Figures and Tables

**Table 1:**
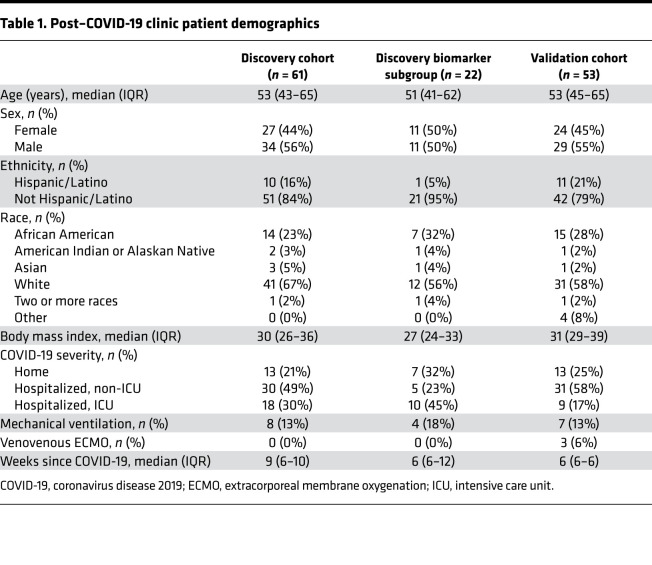
Post–COVID-19 clinic patient demographics

## Version 1 06/10/2021

In-Press Preview

## Version 2 07/22/2021

Electronic publication
